# Impact of thermal manipulation during embryogenesis on thermotolerance and semen quality of Mandarah roosters exposed to heat stress

**DOI:** 10.14202/vetworld.2024.1311-1317

**Published:** 2024-06-19

**Authors:** Ali El-Prollosy, Ebtsam Iraqi, Nadia Elsayed, Hanaa Khalil, Amina El-Saadany, Karim El-Sabrout

**Affiliations:** 1 Department of Poultry Breeding Research, Animal Production Research Institute, Agriculture Research Center, Giza, Egypt; 2 Department of Poultry Production, Faculty of Agriculture, Alexandria University, Alexandria, Egypt

**Keywords:** antioxidant capacity, immunity, incubation temperature, semen characteristics, testosterone, thermotolerance

## Abstract

**Background and Aim:**

The management of incubation conditions impacts embryonic development, hatchability, and post-hatch performance. This study aimed to examine the effects of thermal manipulation (TM) during embryonic development on roosters’ thermotolerance, antioxidant activity, immunity, and semen quality under heat-stress conditions.

**Materials and Methods:**

1200 fertile eggs were distributed evenly between two groups, each containing three replicates (200 eggs/replicate). The first group (G1) was held in the commercial setter with a consistent temperature of 37.5°C and 55% relative humidity (RH) through the 18-day incubation period, acting as a control, while the second group (G2) experienced these conditions until only the 11^th^ day. The eggs were incubated at 39.5°C with 60% RH for 4 h each day from the 12^th^ to the 18^th^ day. From the 19^th^ to 22^nd^ incubation days, both groups maintained a consistent temperature of 37.2°C with a RH of 70%. Two hundred hatched male chicks per treatment group were moved into a closed-system house. All roosters were exposed to a 6-h daily heat challenge with a temperature of 35°C and a humidity of 70% between their 36^th^ and 40^th^ weeks of age.

**Results:**

Roosters of G2 exposed to thermal challenge showed improvements (p ≤ 0.05) in multiple blood biochemical, antioxidant, and immunity markers, including total protein, globulin, aspartate aminotransferase, alanine aminotransferase, triiodothyronine, thyroxine, corticosterone, testosterone, total antioxidant capacity, malondialdehyde, immunoglobulin G, immunoglobulin M, and immunoglobulin A levels. Improved semen quality characteristics, including ejaculate volume, sperm concentration, motility, livability, and quality factor, as well as enhanced thermoregulation in post-hatch cocks, were also achieved (p ≤ 0.05).

**Conclusion:**

To boost antioxidant activity, immunity, thermotolerance, and semen parameters in roosters under heat-stress conditions, TM application during egg incubation, specifically at 12–18 days, is recommended.

## Introduction

Embryonic development, hatchability, chick quality, and post-hatch performance are significantly impacted by incubation conditions [1, 2]. Birds’ physiological and biological features are significantly influenced by temperature [3, 4]. Embryonic thermal manipulation (TM) enhances chick muscle development, efficiency, antioxidant properties, immunity, and decreases mortality rates, according to several studies [4–7].

Heat stress significantly decreases profitability in commercial poultry production by negatively impacting growth rate, welfare, and increasing mortality percentage. Global warming has posed considerable challenges to chicken farming recently. In chickens, thermal stress causes oxidative damage that negatively impacts their physiological performance, including aspects such as cell cycle, enzyme regulation, and metabolic processes [8–10]. Researchers and producers need to persistently search for improved methods to tackle these detrimental alterations. Thermally manipulated fertile eggs during incubation (a critical developmental period for embryos), especially from the 12^th^ to the 18^th^ days, have been shown to improve growth performance and reduce the negative impacts of post-hatch heat stress by enhancing thermotolerance acquisition [2, 4]. Early heat acclimation during pre- or postnatal development can epigenetically regulate gene expression and create long-term physiological memory for improved thermotolerance [2, 11, 12].

The TM procedure during embryogenesis enhances post-hatch performance, especially under hot conditions [10, 13, 14]. In tropical and subtropical regions, roosters’ fertility and semen quality are significantly influenced by temperature. Males, not females, are primarily responsible for poor fertility in chicken breeders raised at temperatures above 22°C [15]. Semen quality deteriorates during the summer because of the negative effects of high ambient temperatures on spermatogenesis [16]. Shanmugam *et al*. [13] investigated the influence of a daily 3-h exposure to 40.5°C and 65% relative humidity (RH) from the 15^th^ to 17^th^ day of hatching on mitigating the detrimental effects of heat stress in roosters. In hot climates, embryonic TM influences the development of reproductive tissue in roosters, leading to enhanced semen parameters.

Although several studies have shown conflicting results regarding TM’s effect on poultry productive performance during embryogenesis [5, 7, 9, 14, 17–19], research is lacking on its impact on the thermotolerance and reproductive capacity of male chickens. The present experiment aimed to explore how TM during embryogenesis impacted thermotolerance and semen quality in Mandarah roosters under heat stress.

## Materials and Methods

### Ethical approval

The experimental procedure was performed in accordance with the guidelines of animal welfare of the European Parliament (2010/63/EU) on the protection of animals used for scientific purposes and the Animal Production Research Institute’s experimental animal care and in accordance with the Experimental Animal Care Committee Ethics of Alexandria University (Alex. Agri. 092312311).

### Study period and location

This study was conducted in the summer (16 July to 15 August) of 2023 at El-Sabahia Poultry Research Station, Animal Production Research Institute, Alexandria (31.2001° N, 29.9187° E).

### Experimental design

A total of 1200 fertile eggs from Mandarah breeder chickens (an Egyptian-improved dual-purpose strain) were randomly and evenly divided into two groups of three replicates (200 eggs each). Each group was incubated in a separate incubator (S380, PTO Company^®^, Egypt) under identical specifications and conditions. The first group (G1) was subjected to a commercial setter temperature of 37.5°C with 55% RH throughout the incubation period (1–18 days) and served as a control, while the second group (G2) was treated with the same commercial setter conditions until the 11^th^ day of incubation. The eggs were then exposed to a higher temperature of 39.5°C with 60% RH for 4 h daily from the 12^th^ to the 18^th^ day of incubation. All eggs in both groups were exposed to the same temperature condition of 37.2°C with 70% RH and an egg turning rate of 6 times/day with a ventilation rate of 350 m³/h from the 19^th^ to the 22^nd^ days of incubation.

Two hundred hatched male chicks per treatment group were transferred from the incubator into a closed-system house (33°C temperature; 60% RH; 15 birds/2.4 m^2^ density; 20 lux light intensity) and randomly distributed into four pens (50 chicks/replicate). They were housed under environmentally controlled light (16L: 8D). At the 36^th^ week of age, all cocks experienced a 6-h heat challenge each day at 35°C with 70% RH between 10:00 a.m. and 16:00 p.m., followed by a return to normal conditions (26°C) within an hour. The thermos challenge lasted until the 40^th^ week of the bird’s life. The birds were given unlimited access to food and water during the experiment.

### Data collection

#### Hematological and biochemical parameters

At the 40^th^ week of age, 60 roosters from each group were chosen at random to collect blood samples for hematological and biochemical analyses. The white blood cell count (WBCs) and their differentiation (monocyte, eosinophil, lymphocyte, and heterophil) were the hematological parameters determined according to El-Saadany *et al*. [20]. The total protein concentration (g/dL) was measured according to Henry *et al*. [21], and the albumin concentration (g/dL) was estimated using a specific commercial kit (Diamond Diagnostics Chemical Company, Egypt). The globulin concentration (g/dL) was calculated by subtracting the total protein from the albumin. Liver enzyme activity (aspartate aminotransferase [AST] and alanine aminotransferase [ALT]) was assayed in the plasma using a specific kit (Diamond Diagnostics Chemical Company, Egypt) procedure. Plasma triiodothyronine (T_3_) and thyroxine (T_4_) levels were analyzed using radioimmunoassay kits (Diagnostic Systems Laboratories, USA). Plasma corticosterone levels were measured using a corticosterone competitive enzyme-linked immunosorbent assay (ELISA) kit (Bioassay Technology Laboratory, China), as described by the manufacturer, and testosterone concentrations were assayed with ELISA, according to the manufacturer’s instructions. Plasma total antioxidant capacity (TAC) and malondialdehyde were determined according to Benzie and Strain [22] and Placer *et al*. [23], respectively. The plasma immunoglobulin G (IgG) concentration was determined using a chicken IgG ELISA kit (CEA544Ga, Enzyme-linked Immunosorbent Assay Kit, Cloud-Clone Corp., USA), and immunoglobulin M (IgM) was assessed using a chicken IgM ELISA kit (Immunology Consultants Laboratory, Inc., USA), according to the manufacturer’s instructions. Immunoglobulin A (IgA) concentration was determined using a chicken IgA ELISA kit (Cloud-Clone Corp., USA).

### Semen collection

For 4 weeks, roosters were prepared for semen collection. 40 roosters’ semen samples were collected twice weekly, throughout the 36–40-week period of the experiment, from both groups randomly. Semen was extracted from cocks by massage of the back and abdomen followed by suction into a sterile tube. During semen collection, the tube was kept in an insulated block to preserve the suitable temperature. 2 h after heat exposure, individual semen samples were collected from cocks for evaluation.

### Semen physical evaluation

Ejaculate volume (mL) was measured using a graduated tube to the nearest 0.01 mL, while sperm concentration (×10^9^ sperm/mL) was estimated using a hemocytometer. Sperm motility was evaluated microscopically at 400× magnification, according to Abioja *et al*. [15]. The percentage of live-dead sperm was determined using a thin smear of semen mixture and Eosin-Nigrosin solution. 400× magnification was used to examine the stained smears for counting. Live sperm was identified as those without stain and dead sperm as those with stain. The semen quality index was determined by multiplying the ejaculate volume (mL), the sperm concentration (10^9^/mL), and the livability (%). The pH of each semen sample was measured right away using a Micro pH Meter (Micro pH Meter, Portugal).

### Statistical analysis

Data were statistically analyzed using the general linear model procedure in accordance with the SAS program (Version 15.1 2018, USA). All values are presented as least-square means with a standard error of the mean. Significant differences between the two groups were subjected to the t-test. Results were considered significant at p ≤ 0.05.

## Results

In general, TM at 12–18 days of egg incubation positively affected several studied traits. It improved semen quality characteristics, such as semen volume and sperm concentrations, as well as some blood biochemical parameters, including antioxidant and immunity indices, resulting in enhanced post-hatch cock’s thermoregulation and reproductive performance.

The effect of TM during egg incubation on some hematological and blood biochemical traits of roosters exposed to thermal challenge is presented in Table-1. TM significantly (p ≤ 0.05) affected WBCs, heterophil (%), H/L ratio, total protein, and globulin concentrations. Treated roosters (G2) had significantly higher (p = 0.013) IgG, IgM, and IgA levels and lower (p = 0.001) AST and ALT levels than G1 (control). Furthermore, G2 showed higher (p = 0.001) TAC and lower (p = 0.001) malondialdehyde than G1. The treated group also had higher (p = 0.001) T_3_, T_4_, and testosterone levels and lower (p = 0.001) corticosterone levels than G1.

**Table-1:** The effect of thermal manipulation at 12–18 days of incubation on some hematological and blood biochemical traits of roosters exposed to thermal challenge.

Traits	G1 (Control)	G2 (TM)	p-value
WBCs (×10^3^/mm^3^)	21.46^b^ ± 0.35	23.81^a^ ± 0.92	0.018
Monocyte (%)	6.86 ± 0.29	7.45 ± 0.26	0.079
Eosinophil (%)	9.96 ± 0.18	9.77 ± 0.18	0.444
Lymphocyte (%)	40.96 ± 0.96	42.61 ± 1.05	0.312
Heterophil (%)	25.72^a^ ± 0.91	21.20^b^ ± 0.94	0.003
H/L ratio	0.63^a^ ± 0.39	0.50^b^ ± 0.32	0.021
Total protein (g/dL)	5.14^b^ ± 0.16	6.24^a^ ± 0.13	0.001
Albumin (g/dL)	3.65 ± 0.05	3.78 ± 0.05	0.068
Globulin (g/dL)	1.51^b^ ± 0.13	2.48^a^ ± 0.13	0.022
IgG (mg/dL)	3.62^b^ ± 0.13	4.18^a^ ± 0.16	0.014
IgM (mg/dL)	0.89^b^ ± 0.01	1.25^a^ ± 0.04	0.012
IgA (mg/dL)	0.02^b^ ± 0.01	0.61^a^ ± 0.11	0.000
AST (U/L)	58.23^a^ ± 1.89	41.79^b^ ± 0.40	0.000
ALT (U/L)	19.91^a^ ± 0.38	16.83^b^ ± 0.06	0.001
TAC (mMol/dL)	385.94^b^ ± 5.92	441.37^a^ ± 4.93	0.000
Malondialdehyde (nmol/mL)	1.17^a^ ± 0.06	0.88^b^ ± 0.03	0.003
T_3_ (ng/mL)	2.83^b^ ± 0.07	3.88^a^ ± 0.09	0.001
T_4_ (ng/mL)	14.76^b^ ± 0.52	17.87^a^ ± 0.15	0.001
Corticosterone (ng/mL)	25.31^a^ ± 1.24	18.85^b^ ± 1.03	0.003
Testosterone (ng/dL)	427.92^b^ ± 3.31	446.86^a^ ± 1.36	0.001

^a,b^Means having different letters in the same row are significantly different (p ≤ 0.05). TM=Temperature manipulation, H/L ratio=Heterophils/lymphocytes ratio. n = 60 blood samples from each group, WBCs=White blood cell, IgG=Immunoglobulin G, IgM=Immunoglobulin M, AST=Aspartate aminotransferase, ALT=Alanine aminotransferase, TAC=Total antioxidant capacity

Furthermore, TM during embryogenesis had a positive influence (p ≤ 0.05) on the semen quality parameters of roosters exposed to thermal challenge (Table-2). TM significantly (p ≤ 0.05) affected the ejaculate volume, sperm concentration, mass motility, sperm livability, and semen quality factor, but it did not affect (p = 0.647) the semen pH value. G2 roosters had significantly higher (p ≤ 0.001) ejaculate volume, sperm concentration, sperm livability, and lower (p ≤ 0.001) sperm mortality than G1. Moreover, G2 had significantly higher (p ≤ 0.001) mass motility and semen quality factor than G1.

**Table-2:** The effect of thermal manipulation at 12–18 days of incubation on semen quality parameters of roosters exposed to thermal challenge.

Parameters	G1 (Control)	G2 (TM)	p-value
Ejaculate volume (mL)	0.51^b^ ± 0.04	0.64^a^ ± 0.03	0.002
Sperm concentration (×10^9^/mL)	2.43^b^ ± 0.09	2.99^a^ ± 0.15	0.005
Mass motility (%)	82.39^b^ ± 1.88	89.53^a^ ± 1.20	0.001
Sperm livability (%)	81.73^b^ ± 1.49	92.40^a^ ± 1.03	0.000
Sperm mortality (%)	18.26^a^ ± 1.49	7.60^b^ ± 1.03	0.001
Semen quality factor	1.01^b^ ± 0.09	1.77^a^ ± 0.14	0.000
Semen pH value	7.24 ± 0.04	7.21 ± 0.01	0.647

^a,b^Means having different letters in the same row are significantly different (p ≤ 0.05). TM=Temperature manipulation. n = 40 semen samples from each group

## Discussion

Birds primarily experience stress due to temperature fluctuations. The summer temperature can surpass 35°C in certain tropical and subtropical nations. Birds are highly susceptible to heat stress without sweat glands and limited thermoregulatory ability. Developing a strategy to enhance thermotolerance and manage heat stress in post-hatch life is crucial. The current study represents the second part of a previously published study [4] entitled “Effect of thermal manipulation on embryonic development, hatching process, and chick quality under heat-stress conditions”. By manipulating the embryo’s environment during incubation (on days 12–18), we explored the potential of this method to improve thermoregulation, antioxidant protection, immunity, and reproductive performance in roosters facing heat stress.

Blood tests can indicate both an animal’s health and its productivity. They can identify metabolic changes and evaluate an animal’s overall internal physiological processes [20, 24]. According to the current study, roosters’ TM usage during incubation improved hematological and blood biochemical traits, namely WBCs (11%), heterophil (17.5%), H/L ratio (20.5%), total protein (21%), and globulin (64%) concentrations under thermal stress. Compared to the control (G1), roosters in group G2 displayed increased IgG, IgM, and IgA levels as well as decreased AST and ALT levels. G2 had a 14% increase in TAC and a 24.5% decrease in malondialdehyde levels compared to G1. Compared to G1, the treated group exhibited increased T_3_ (37%), T_4_ (21%), and testosterone (4.5%) levels and decreased corticosterone (25.5%) levels. At high ambient temperatures, roosters’ antioxidant capacity, immunity response, and thermoregulation can be positively improved by TM.

According to Table-1, the treated roosters (G2) experienced a significant enhancement in lymphocyte percentage and reduction in heterophil percentage due to TM. Long-term health and resilience to stress are positively correlated with the number of lymphocytes in the peripheral circulation [25]. Christensen *et al*. [26] and Iraqi *et al*. [4] found that incubation temperature affects post-hatch performance quality by altering hormone concentrations (such as T_3_ and T_4_) related to the embryo’s metabolism and growth. Changes in the hypothalamus-pituitary threshold level might explain alterations in some blood biochemical features. Previous studies by Han *et al*. [5] and Piestun *et al*. [27] have shown that elevated temperatures can enhance catalytic activity and respiration in birds, boosting their overall physiological performance. The hypothalamus, hormonal control center in birds, can be influenced by TM, leading to changes in organogenesis and overall performance [8, 28–30]. The impact of TM on corticosterone and other hormones could represent an epigenetic temperature adaptation due to the identical mechanisms being used for managing post-hatch heat stress [10, 31, 32]. The precise phase, frequency, duration, and amplitude of TM are crucial for generating epigenetic adaptations [19, 32].

The studies by Iraqi *et al*. [4], Zaboli *et al*. [14], and Elsayed [33] revealed that embryonic TM positively influences birds’ blood biochemistry, specifically increasing total protein, albumin, T3, and corticosterone levels. The hematological and biochemical changes may be caused by TM exposure during embryonic development affecting the liver and Fabricius gland [4, 5, 34]. Enhancing antioxidant activity in cell membranes is a crucial function of TM for overall bodily processes [9, 35]. Early TM treatment effectively recovers normal biochemical parameters, restores liver function, and boosts antioxidant and immunological status in birds exposed to thermal challenge.

The growth conditions and functional status of immune organs during embryogenesis significantly influence systemic post-hatch bird thermoregulation and immunity. Recent studies have proposed that pre- or post-hatch TM might improve the long-term thermotolerance of birds by reducing body temperature [4, 14, 30]. Han *et al*. [5] and Ramiah *et al*. [12] showed that TM application during chicken egg incubation enhances birds’ thermoregulation and mitigates negative consequences of high ambient temperatures. Ouchi *et al*. [36] found that thermal conditioning in neonatal birds enhances thermotolerance and reduces subsequent body temperature rises upon exposure to high temperatures. Transcranial magnetic stimulation (TM) can enhance birds’ stress response adaptability by altering their adrenal threshold responses [12, 31]. During embryogenesis, TM implementation can enhance the development of the bird’s immune organs, including the thymus and bursa [8, 17]. During the early growth stage, some biochemical alterations, like those affecting thyroid hormones, can result in a long-term shift in an organism’s thermotolerance threshold in response to high environmental temperatures [37]. Al-Zghoul *et al*. [7] found that embryonic TM heat exposure enhances oxidative stress response, tissue stability, and immunological response to heat stress.

It is crucial to understand how TM influences chicken embryos’ embryos’ hypothalamus and the correlation with heat shock protein (HSP) induction in thermotolerance development. The heat shock response involves increased production of cytoprotective HSP70 and reduced cytokine levels upon repeated exposure to heat, leading to lower indicators of cellular and systemic heat strain [12, 38]. HSP70, a prominent liver HSP, shields cells and tissues from heat stress damage and stimulates the secretion of various inflammatory cytokines [39]. According to Al Amaz *et al*. [2], TM promotes the expression of certain HSPs’ genes, aids in thermoregulation, shields the metabolically active embryonic liver from cellular damage, lowers apoptosis during embryogenesis, and contributes to a lower embryo mortality rate. HSP mRNA expression in certain muscles significantly increased due to different TM protocols [12, 40, 41], and this has been associated with improved thermoregulation and thermotolerance, according to Al-Aqil and Zulkifli [42]. However, Ramiah *et al*. [12] reported that TM during embryogenesis has been shown to improve birds’ heat tolerance and well-being without compromising their future performance potential. Based on the previous findings, TM can be a potent practical strategy employed during egg incubation to improve chicks’ physiological, antioxidative, and immunological states, resulting in many beneficial effects on post-hatch chick performance.

Shanmugam *et al*. [13] proposed that heat exposure could potentially cause nuclear abnormalities, thereby decreasing fertility. Elevated free radical levels, primarily in the form of reactive oxygen species (ROS), can lead to both heat stress-induced reductions in the number of penetrating sperm and semen oxidative stress [43, 44]. Chicken sperm has polyunsaturated fatty acids prone to oxidation by free radicals. Elevated free radical levels harm various sperm structures. The proper functioning of acrosome reactions and fertilization necessitates the presence of small quantities of ROS, according to Abioja *et al*. [15]. The experimental data (Table-2) reveal that TM during embryogenesis enhances semen quality parameters in roosters exposed to thermal stress. The intervention significantly improved sperm volume by 25.5%, concentration by 23%, motility by 8.5%, livability by 13%, and semen quality by 75% than control. The study of Shanmugam *et al*. [13] reveals that TM during embryogenesis enhances semen parameters, including semen volume and live sperm, in adult roosters facing hot climates, in agreement with our findings. The long-term benefits of embryogenesis TM on roosters’ antioxidant capacity and testosterone levels could explain these improvements. Zaboli *et al*. [14] reported long-term benefits for male broilers regarding physiological responses from TM during thermal regulation development, which mitigated the negative impacts of chronic heat stress and improved thermotolerance. Early TM application lessens heat stress effects on roosters and enhances their reproductive performance under hot environmental conditions.

## Conclusion

This study is the first to examine the impact of a 4-h daily increased incubation temperature of 39.5°C with 60% RH from the 12^th^ to 18^th^ days on roosters’ thermotolerance, immunity, and semen quality during thermal stress. Based on the results, roosters show enhanced blood biochemical, antioxidant, and immunity markers after early thermal treatment. The roosters’ adaptation to heat stress and semen quality were also improved by it. Applying TM at 12–18 days during egg incubation enhances roosters’ antioxidant activity, immunological response, thermotolerance, and semen quality under heat-stress conditions.

## Data Availability

The supplementary data can be available from the corresponding author on a reasonable request.

## Authors’ Contributions

AE, EI, and HK: Designed and supervised the study. AE, EI, AE, NE, and KE: Performed the experiment and analyzed the data. KE and EI: Wrote the manuscript with approval from all authors. All authors have read, reviewed, and approved the final manuscript.
